# P-TEFb, the Super Elongation Complex and Mediator Regulate a Subset of Non-paused Genes during Early *Drosophila* Embryo Development

**DOI:** 10.1371/journal.pgen.1004971

**Published:** 2015-02-13

**Authors:** Olle Dahlberg, Olga Shilkova, Min Tang, Per-Henrik Holmqvist, Mattias Mannervik

**Affiliations:** 1 Dept. Molecular Biosciences, the Wenner-Gren Institute, Stockholm University, Stockholm, Sweden; 2 Dept. Biochemistry & Biology, South China University, Hengyang, Hunan Province, China; The University of North Carolina at Chapel Hill, UNITED STATES

## Abstract

Positive Transcription Elongation Factor b (P-TEFb) is a kinase consisting of Cdk9 and Cyclin T that releases RNA Polymerase II (Pol II) into active elongation. It can assemble into a larger Super Elongation Complex (SEC) consisting of additional elongation factors. Here, we use a miRNA-based approach to knock down the maternal contribution of P-TEFb and SEC components in early *Drosophila* embryos. P-TEFb or SEC depletion results in loss of cells from the embryo posterior and in cellularization defects. Interestingly, the expression of many patterning genes containing promoter-proximal paused Pol II is relatively normal in P-TEFb embryos. Instead, P-TEFb and SEC are required for expression of some non-paused, rapidly transcribed genes in pre-cellular embryos, including the cellularization gene *Serendipity-α*. We also demonstrate that another P-TEFb regulated gene, *terminus*, has an essential function in embryo development. Similar morphological and gene expression phenotypes were observed upon knock down of Mediator subunits, providing *in vivo* evidence that P-TEFb, the SEC and Mediator collaborate in transcription control. Surprisingly, P-TEFb depletion does not affect the ratio of Pol II at the promoter versus the 3’ end, despite affecting global Pol II Ser2 phosphorylation levels. Instead, Pol II occupancy is reduced at P-TEFb down-regulated genes. We conclude that a subset of non-paused, pre-cellular genes are among the most susceptible to reduced P-TEFb, SEC and Mediator levels in *Drosophila* embryos.

## Author Summary

Embryo development involves formation of various cell types through the regulation of gene transcription, resulting in expression of cell type specific RNAs and proteins. A key regulatory step in transcription of animal genes involves the transition of RNA polymerase II (Pol II) into active elongation. At many genes, Pol II is transiently paused approximately 50 basepairs downstream of the transcription start site. Release from this promoter-proximal pausing involves the kinase P-TEFb, which phosphorylates negative elongation factors, allowing Pol II to enter into productive elongation. In this work, we have depleted a considerable amount of P-TEFb from early *Drosophila* embryos. We find that several genes with paused Pol II can be expressed relatively normally in P-TEFb depleted embryos, whereas expression of some non-paused genes is substantially reduced. This result suggests that also non-paused genes transit through a P-TEFb-dependent checkpoint before entering active elongation. Unexpectedly, we find less Pol II associated with these non-paused genes in P-TEFb embryos. We demonstrate that a protein complex involved in recruitment of Pol II to promoters, the Mediator complex, show the same morphological and gene expression phenotypes as P-TEFb. We propose that Mediator and P-TEFb function together in recruiting Pol II to a subset of developmental genes.

## Introduction

RNA Polymerase II (Pol II) is responsible for the transcription of protein-coding genes in eukaryotes, and differs from all other RNA Polymerases in that its largest subunit contains a C-terminal domain (CTD) consisting of a heptapeptide repeat with the consensus YSPTSPS [reviewed in 1]. Phosphorylation of the CTD is linked to various steps in the transcription cycle. Hypophosphorylated Pol II is recruited to the transcription start site (TSS) and forms a pre-initiation complex. Phosphorylation of Ser5 in the heptapeptide repeat is coupled to initiation of transcription. The transition into transcription elongation involves CTD Ser2 phosphorylation. CTD phosphorylation also links transcription with RNA processing, since the CTD forms a platform for the assembly and action of enzymes involved in capping (Ser5 phosphorylated CTD), splicing and polyadenylation (Ser2 phosphorylated CTD) [reviewed in 2].

Genome-wide mapping of Pol II has shown that it pauses around 50 bp downstream of the TSS at a majority of genes in mammalian and *Drosophila* cells [3,4,5]. Thus, release from promoter-proximal pausing may be a rate-limiting step in the expression of thousands of genes [reviewed in 6]. Indeed, a majority of the genes involved in *Drosophila* embryo patterning are regulated at the level of release from pausing [7]. Paused Polymerase is Ser5 phosphorylated, and release from pausing is associated with Ser2 phosphorylation as well as phosphorylation of the elongation factors DRB sensitivity-inducing factor (DSIF) and negative elongation factor (NELF) [reviewed in 8,9].

Positive transcription elongation factor b (P-TEFb) was purified based on its activity in *Drosophila* nuclear extracts treated with the ATP analog 5,6-dichloro-1-ß-D-ribofuranosylbenzimidazole (DRB) [reviewed in 10]. P-TEFb consists of Cyclin-dependent kinase 9 (Cdk9) and its cyclin partner, Cyclin T (CycT). By phosphorylating the negative elongation factors DSIF and NELF, P-TEFb allows Pol II to enter into productive elongation. The P-TEFb inhibitor flavopiridol affects expression of virtually all genes in *Drosophila* and mouse cells [11,12], showing that release into elongation is a key regulatory event in metazoan gene expression. Active, non-paused genes are most sensitive to P-TEFb inhibition, indicating that they require P-TEFb continuously for rapid release into elongation [11].

A large fraction of P-TEFb is bound to the 7SK snRNP that sequesters the kinase in an inactive state [13]. Another fraction of P-TEFb is found in a protein complex known as the Super Elongation Complex (SEC) [reviewed in 14]. In *Drosophila*, the SEC contains the elongation factor ELL (eleven-nineteen lysine-rich leukemia), the ELL-associated factor Eaf, the ENL/AF9-like protein Ear, the AFF (AF4/FMR2 family)-like protein Lilliputian (Lilli), and P-TEFb [15]. The SEC appears to be particularly important for rapid induction of transcription [16]. The mechanism by which P-TEFb and the SEC are recruited to gene promoters is not understood, but recently the Mediator subunit MED26 has been suggested to be involved [17].

Embryo development in most insects involves cellularization, whereby the nuclei in a syncytium become enclosed by plasma membrane. In *Drosophila melanogaster*, thirteen mitotic divisions occur without cytokinesis [reviewed in 18]. Cellularization is controlled by the maternal to zygotic transition (MZT), where maternal transcripts are destabilized, and zygotic genome activation necessary for the event to initiate [reviewed in 19]. Four zygotically transcribed genes are critical, *Serendipity-α* (*Sry-α*) and *bottleneck (bnk)* are required for stability and organization of microfilaments [20], whereas *nullo* controls formation of endocytic vesicles needed for membrane trafficking [21], and *slow-as-molasses (slam)* localizes RhoGEF2 to the cleavage furrows that enclose the nuclei into cells [22]. Transcription of these genes requires the maternally provided Zinc-finger transcription factor Zelda [23], whereas the SEC component Lilli is necessary for *Sry-α* expression, but not for expression of *bnk* and *nullo* [24].

Recently, it was shown that cellularization genes and other genes transcribed in pre-cellular embryos do not show Pol II pausing, and are associated with distinct core promoter elements compared to genes activated at the major wave of the MZT [25]. Pre-cellular genes are generally short and rapidly transcribed, since the nuclear cycles are only between 8 and 13 min long at this stage and progression through mitosis causes abortion of nascent transcripts [26,27]. Consistent with their fast transcription, pre-cellular genes are shorter and more often intronless than genes activated at the MZT [25].

Previous work showed that P-TEFb depletion in *Drosophila* larvae results in decreased chromatin bound Ser2 phosphorylated Pol II [28], and in wing patterning defects [29]. Similarly, larval knockdown of the P-TEFb regulator dHEXIM (a component of the 7SK snRNP) results in severe leg and wing phenotypes [13]. However, the role of P-TEFb in embryogenesis is poorly understood. Here, we provide evidence for a fundamental role for P-TEFb during early development. Reduced P-TEFb levels do not grossly affect expression of most genes involved in embryo patterning, despite the presence of paused Pol II at many of these genes. Instead, expression of a subset of non-paused, rapidly transcribed genes in pre-cellular embryos is diminished by P-TEFb depletion. The same genes are affected by depleting the SEC and Mediator complexes, providing *in vivo* evidence that P-TEFb, the SEC and Mediator collaborate in the regulation of rapidly transcribed genes. Our results show that some non-paused genes are more reliant on P-TEFb than many paused genes in the early embryo.

## Results

### P-TEFb is required for early *Drosophila* embryo development

**Figure 1:**
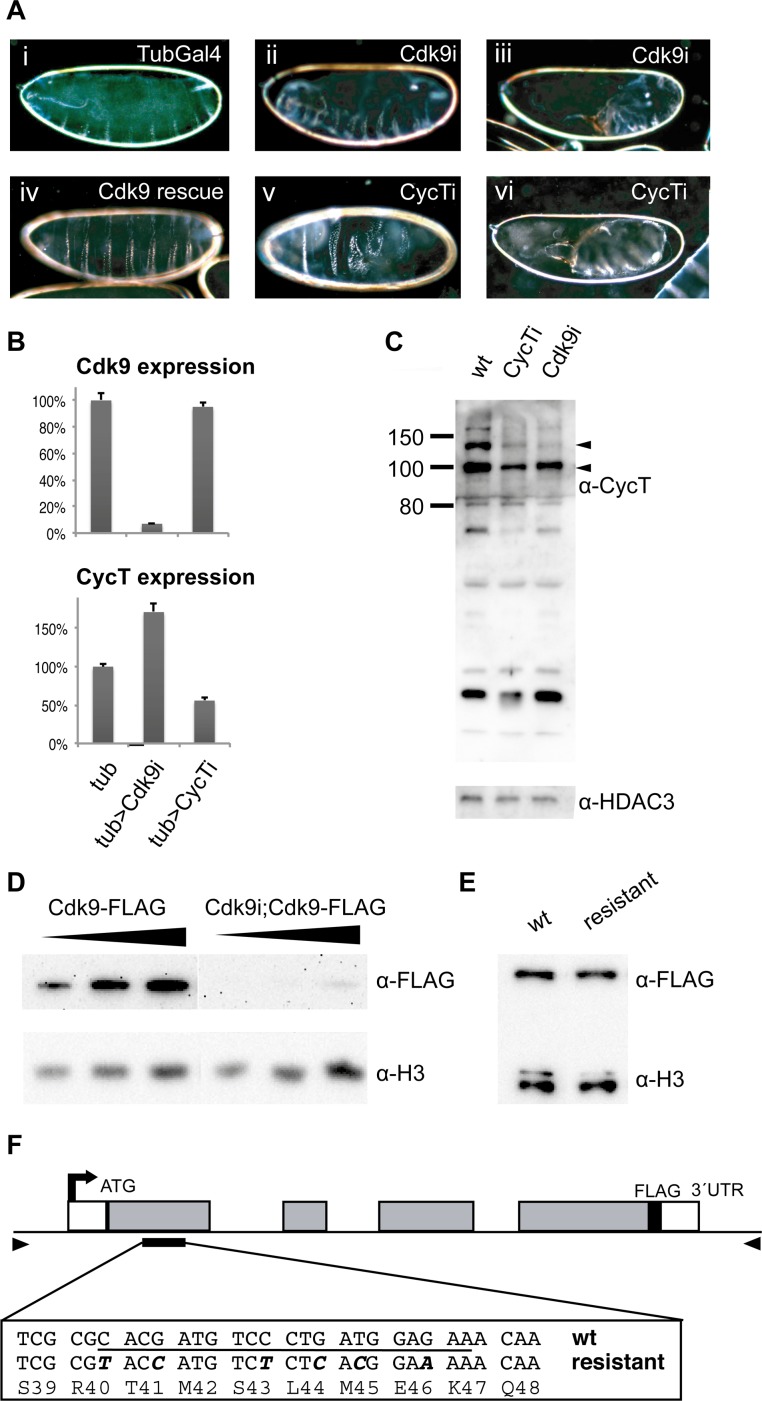
Maternal knockdown of P-TEFb disrupts embryo development. (**A**) Embryos were collected from females containing the maternal α-Tubulin-Gal4-VP16 (TubGal4) driver alone (i), TubGal4 and a shmiRNA targeting Cdk9 (ii, iii) or CycT (v, vi), or TubGal4, Cdk9 shmiRNA and an miRNA-resistant Cdk9 transgene (iv). Dark-field micrographs of embryo cuticle preparations show patterning defects. (**B**) Quantitative RT-PCR of Cdk9 and CycT expression relative four reference genes in 2–4 hour old maternally depleted embryos derived from females with TubGal4 (tub) crossed to wild-type (*w* ^*1118*^, control), or to shmiRNAs targeting Cdk9 or CycT. Expression in control embryos was set to 100%. Error bars denote S.E.M. n = 6. (**C**) Western blot showing CycT levels in wild-type 2–4 h embryos and in embryos depleted of maternal CycT or Cdk9. HDAC3 was used as a loading control. Molecular weight markers are indicated to the left, and arrowheads point to the predicted 118 and 94 kDa CycT isoforms. (**D**) Western blot of FLAG epitope-tagged Cdk9 expressed from its endogenous promoter demonstrates reduced Cdk9 protein in Cdk9 miRNA depleted 2–4 h embryos. Histone H3 was used as a loading control. (**E**) Western blot of FLAG-tagged Cdk9 transgenes in adult flies with either the native DNA sequence or an miRNA resistant sequence shows comparable levels of protein. Histone H3 was used as a loading control. (**F**) A schematic drawing of the *Cdk9* locus. The sequence targeted by the miRNA is underlined, and the nucleotide changes in the miRNA resistant transgene highlighted in bold and italic. The arrowheads indicate the positions of the primers used to amplify the genomic region used in the transgenes.

In order to study the role of P-TEFb in early *Drosophila* embryo development, we depleted its maternal contribution using short hairpin micro RNAs (shmiRNAs) expressed in the female germline [30]. These shmiRNAs are designed to effectively deplete maternal protein in ovaries [31]. We used α-Tubulin-Gal4-VP16 to drive UAS-shmiRNAs in the female germline, and collected embryos from mothers targeting P-TEFb (Cdk9 or CycT, hereafter referred to as Cdk9, CycT, or P-TEFb embryos) and embryos from control mothers expressing the α-Tubulin-Gal4-VP16 transgene alone. Cuticle preparations showed that P-TEFb embryos failed to hatch and produced variable patterning defects (Fig. 1A). We examined the extent of knock-down in 2–4 hour old embryos by RT-qPCR and by Western blotting (Fig. 1B-D). Cdk9 mRNA expression was reduced to 7% of controls in Cdk9 embryos, but was not affected in CycT embryos, whereas CycT mRNA levels were reduced to 56% of controls in CycT embryos, and increased in Cdk9 embryos (Fig. 1B). In wild-type embryos, multiple bands were detected with a CycT antibody, which may represent isoforms generated by alternative splicing as well as breakdown products (Fig. 1C). In CycT embryos, the 118 kDa CycT isoform was reduced to 27% of wild-type and the 94 kDa isoform to 65%. Interestingly, maternal Cdk9 knock-down also resulted in diminished CycT levels (24% and 71% of wild-type, Fig. 1C). This suggests that an interaction between Cdk9 and CycT stabilizes the CycT protein. Since we did not have access to a Cdk9 antibody, we generated a FLAG-tagged Cdk9 protein expressed from its endogenous promoter (Fig. 1F). We crossed this transgene into females expressing the *Cdk9* shmiRNA in the germline, and into females expressing only the α-Tubulin-Gal4-VP16 driver as a control. A Western blot showed a strong reduction of FLAG-tagged Cdk9 protein in embryos from mothers with Cdk9 knock-down compared to the control (3% of control, Fig. 1D), consistent with the effect on Cdk9 mRNA levels (Fig. 1B).

To examine if the P-TEFb phenotype was due to off-target effects, we mutated the transgenic *Cdk9* construct in the miRNA binding site to make a miRNA-resistant gene using synonymous codons already existing in other parts of the gene (Fig. 1F). A FLAG-tagged version of the miRNA-resistant transgene was integrated in the same genomic position as the FLAG-tagged wild-type transgene, and the expression levels compared in adult flies by Western blot (Fig. 1E). No difference in expression was seen, the wild-type transgene and the miRNA-resistant transgene produced a similar amount of protein. This shows that the synonymous codons do not impair translation of Cdk9 protein. However, the FLAG-tagged transgene failed to rescue the *Cdk9* mutant phenotype. We therefore produced a non-tagged miRNA-resistant *Cdk9* transgenic fly and crossed it into females expressing the *Cdk9* shmiRNA in the germline, which rescued the embryonic phenotype and lethality (Fig. 1A and S1 Table). This shows that the embryonic phenotype was due to depletion of P-TEFb, and was not caused by off-target effects. It also demonstrates that the FLAG-tag interferes with Cdk9 function. Taken together, our results suggest that miRNAs targeting P-TEFb efficiently and specifically reduce P-TEFb protein levels and disrupt embryo development.

### Maternal P-TEFb depletion results in loss of cells from the embryo posterior and in cellularization defects

**Figure 2:**
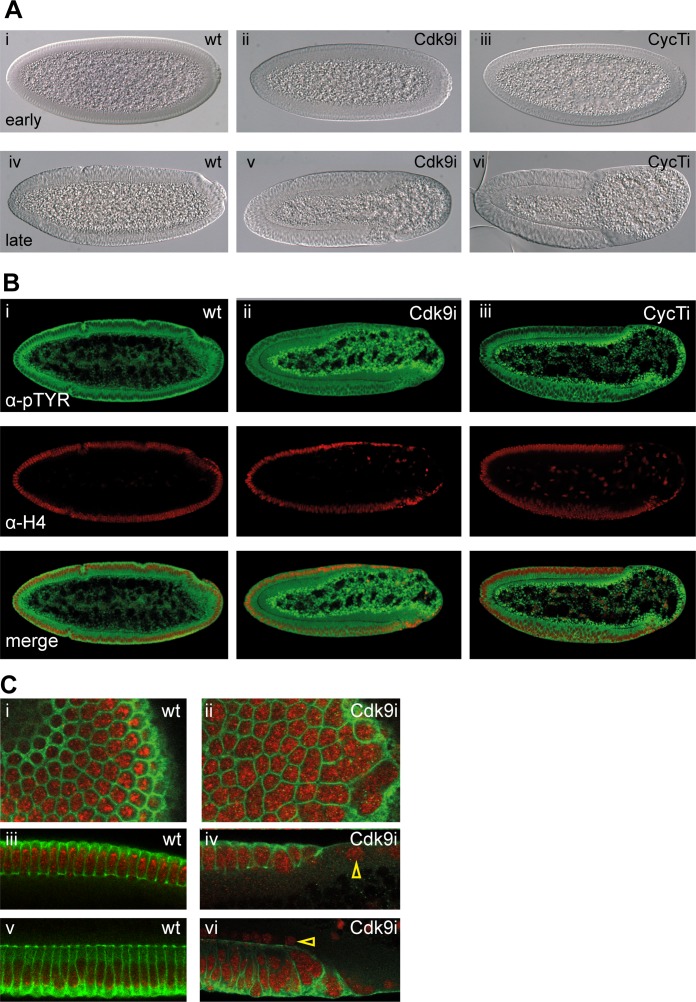
P-TEFb embryos show cellularization defects and lose cells in the embryo posterior. (**A**) Differential interference contrast (DIC) microscopy reveals a posterior loss of cells that appear prior to gastrulation in P-TEFb depleted embryos. Cellularizing P-TEFb embryos look normal (ii and iii), whereas cellularized embryos are missing cells in the posterior (v and vi). Compare to wild-type (wt) embryos in (i) and (iv). (**B, C**) Confocal images of wild-type (Bi, Ci, iii, v) and P-TEFb embryos immunostained with anti-phosphotyrosine (pTYR) antibody that marks the plasma membrane (green) and anti-Histone 4 marking the nuclei (red). Cells are lost from the posterior in P-TEFb embryos (Bii and iii). In addition, cell shape changes as well as multi-nucleated cells are evident in Cdk9 depleted embryos (Cii), the nuclei do not elongate as in wild-type (compare Ciii with iv), and some nuclei are present outside the basal side of cells in Cdk9 embryos (yellow arrowheads in C iv and vi). In cellularized P-TEFb embryos, mesodermal cells are not properly arranged (Bii, iii, and C vi). Embryos were generated as in Fig. 1, and are oriented with anterior to the left and dorsal side up in A and B.

Maternal knock-down of P-TEFb resulted in a posterior-specific loss of cells in embryos prior to gastrulation (Fig. 2). Differential interference contrast (DIC) microscopy showed embryos with normal morphology in the syncytial blastoderm stage (Fig. 2Aii and iii), with the phenotype appearing in cellularized embryos, where a substantial part of the embryo posterior was missing cells (Fig. 2Av and vi). Gastrulation occurred but was delayed, as the cephalic and ventral furrows were formed later than in wild-type. Depletion of Cdk9 or CycT resulted in undistinguishable phenotypes, strongly indicating that the two subunits of P-TEFb function together in the embryo.

In order to follow when nuclei disappear from the posterior, we used live imaging with RFP-tagged histone H2Av. Time-lapse movies of Cdk9 depleted embryos showed that the nuclei migrated to the cortex in the entire embryo during division cycle 10 (S2 Movie), as in wild-type embryos (S1 Movie). The posterior phenotype originated from an abnormal loss of posterior nuclei from the cortex prior to germ-band extension. Even though no cells remained in the posterior after this migration, Cdk9-depleted embryos still underwent gastrulation. However, germ band extension did not proceed normally. Sporadic folds appeared and twisting of the embryo was seen in many cases (S2 Movie).

Wild-type and P-TEFb embryos were stained with a phosphotyrosine antibody, which labels the cell membrane, and with a histone H4 antibody to label the nuclei (Fig. 2B and C). In addition to missing cells in the posterior (Fig. 2B), defects in cell shape as well as multinucleated cells were found in P-TEFb embryos (Fig. 2C). Another feature of P-TEFb embryos was that nuclei and cells were not aligned laterally in the mesoderm, but were occasionally located on top of other cells. Nuclei could also be found outside of the basal cell membranes after cellularization (Fig 2C). This shows that cellullarization is perturbed in P-TEFb embryos.

### *Serendipity-α* gene expression is controlled by P-TEFb and the Super Elongation Complex

**Figure 3:**
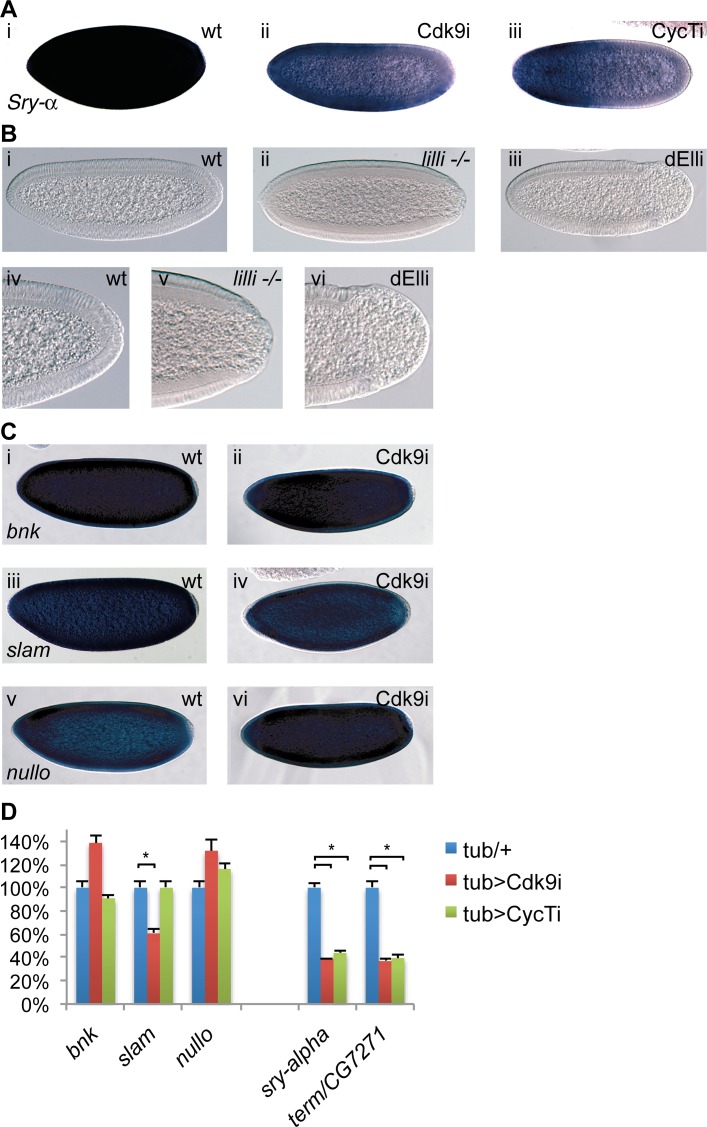
P-TEFb and the Super Elongation Complex (SEC) display similar phenotypes. (**A**) RNA *in situ* hybridization using a digoxigenin-labeled *Serendipity-α (Sry-α)* probe demonstrates decreased levels of *Sry-α*mRNA in embryos depleted of maternal Cdk9 (ii) or Cyclin T (iii) compared to wild-type (i). (**B**) DIC micrographs of wild-type (wt, i), or embryos derived from females with germline clones of the SEC component dAFF4/Lilliputian (*lilli -/-*, ii) or females expressing TubGal4 and a shmiRNA targeting the SEC subunit dEll (dELLi, iii) show the same posterior phenotype as in P-TEFb embryos. A close up of the same embryos is shown below (iv-vi). (**C**) Expression of the cellularization genes *bottleneck* (*bnk*, i, ii), *slow-as-molasses* (*slam*, iii, iv), and *nullo* (v, vi) is comparable between wild-type and embryos depleted of maternal Cdk9. (**D**) Quantification of cellularization gene expression by RT-qPCR. Columns show average values in 2–4h embryos with S.E.M. (n = 5–6) and control values were set to 100%. Relative expression was normalized to a mean of four reference genes (*beta-tubulin*, *GAPDH*, *RpL32*, and *28SrRNA*). *indicates P<0.05, two-tailed paired Student’s t-test.

To further explore the cellularization phenotype, we examined expression of genes involved in this process by RNA *in situ* hybridization. Knock-down of either Cdk9 or CycT resulted in a reduction in *Serendipity-α* (*Sry-α)* mRNA abundance (Fig. 3A). Since a component of the super elongation complex (SEC), Lilliputian (Lilli), has previously been shown to activate *Sry-α*expression [24], depletion of the SEC shows similar gene expression changes as P-TEFb during early development. Therefore, we investigated if the posterior phenotype could also be seen in *lilli* mutant embryos. We generated embryos lacking maternal Lilli by inducing germ-line clones with the strong *lilli* ^*XS575*^ allele [24]. Although this phenotype was not previously reported, we observed loss of posterior cells in *lilli* mutant embryos (Fig. 3B). The cell-depleted area was somewhat smaller in *lilli* than in P-TEFb depleted embryos (compare Fig. 3B with Fig. 2A). We next examined if another component of the SEC complex showed the same phenotype. We used a shmiRNA targeting the elongation factor *dELL* [32] to knock-down maternal dELL protein. Offspring derived from these mothers also showed loss of cells from the embryo posterior (Fig. 3B).

To find more genes that are sensitive to P-TEFb or SEC depletion, we examined a subset of genes that have a very rapid induction during the maternal to zygotic transition (MZT), since the SEC is known to be involved in rapid induction of genes [16]. We manually selected a set of genes that peak in zygotic expression during 2–4 h of development, but had low or no transcript levels in 0–2 hour old embryos (none or little maternal transcript, Flybase data [33]). Several genes involved in cellularization were found, including *Sry-α*, *bnk*, *slam* and *nullo*. We investigated the expression of these genes in Cdk9 depleted embryos by *in situ* hybridization, but did not observe reduced mRNA levels of *bnk*, *slam*, or *nullo*, suggesting that they are activated by a mechanism not sensitive to reduced P-TEFb levels (Fig. 3C). Similarly, embryos derived from *lilli* germline clones were previously demonstrated to downregulate *Sry-α*, but showed no change in the protein levels of Nullo and Bnk [24]. We also used quantitative RT-PCR to measure the mRNA levels of these cellularization genes in Cdk9 and CycT embryos, which largely confirmed the results obtained by *in situ* hybridization (Fig. 3D). We observed small changes in *bnk*, *slam* and *nullo* expression in Cdk9 and CycT embryos, although *slam* expression was reduced in Cdk9 but not in CycT embryos (Fig. 3D). By contrast, *Sry-α* expression was reduced by both Cdk9 and CycT knock-down (Fig. 3D), as was also observed by *in situ* hybridization (Fig. 3A). This shows that reducing the levels of P-TEFb and the SEC is of little consequence for the expression of several genes in the early embryo and selectively affects gene expression.

### *Terminus* is a P-TEFb-sensitive gene that is essential for embryo development

**Figure 4:**
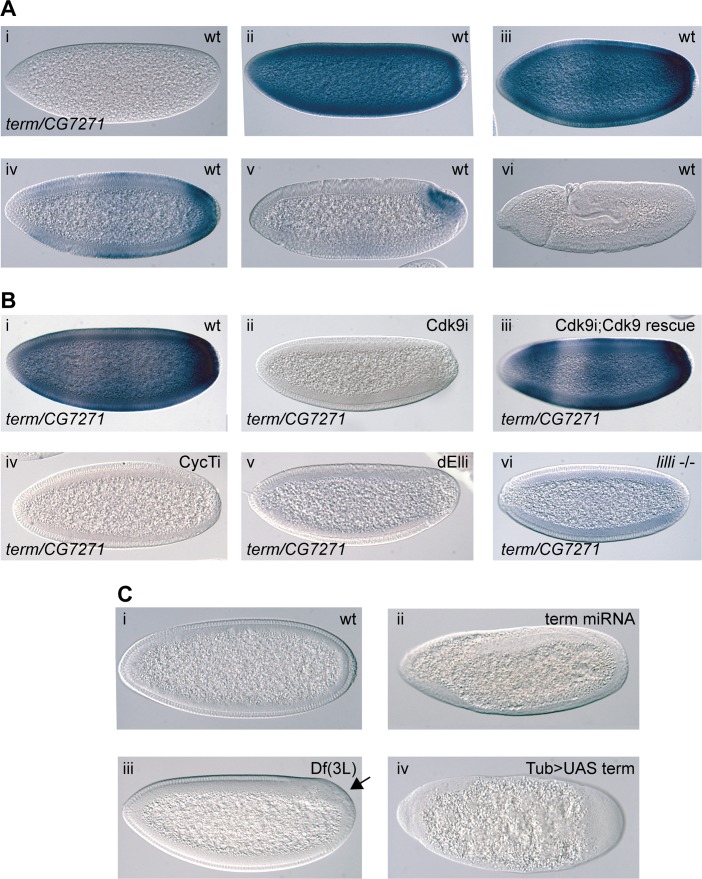
The P-TEFb and SEC-regulated gene *terminus (term)* is essential in early embryos. (**A**) *In situ* hybridization showing *term/CG7271* expression in wild-type during different embryo stages (i-vi). The transcripts become concentrated to the posterior in cellularized embryos (iv and v) (**B**) Expression of *term/CG7271* is severely reduced in embryos depleted of maternal Cdk9 (ii) compared to wild-type (i), and rescued by the miRNA-resistant transgene in Cdk9 embryos (iii). Greatly diminished *term/CG7271* levels are also observed in embryos depleted of maternal CycT (iv) or dEll (v), and in embryos from *lilli* germline clones (vi). (**C**) Knockdown of zygotic *term* by crossing TubGal4 females with Term shmiRNA males (ii), eliminating Term and CG7271 by deletion in embryos derived from the deficiency *Df(3L)BSC416* (iii), or over-expressing Term by crossing TubGal4 females with UAS-*term* males (iv) results in severe morphological defects in early embryos, including a failure to form a cellular blastoderm. A wild-type cellularizing embryo (i) is shown for comparison.

Among other genes having a rapid zygotic expression, we focused on the gene *terminus*. Its mRNA is not maternally provided, but the gene is activated at high levels throughout the embryo during the MZT, with the highest expression in the most posterior part of the embryo (Fig. 4A and [34]). Expression then rapidly decreases in most cells until the mRNA is only localized to the posterior pole, just prior to germ band extension (Fig. 4A and [34]). A BLAST search showed that the coding DNA sequence of *term* is 99.1% homologous to the gene *CG7271*. Since *CG7271* is located next to the *term* locus in a head to head orientation, it is likely the result of a recent gene duplication. Given the high sequence similarity between *CG7271* and *term*, we did not expect the *term* probe to distinguish between the two gene products. We therefore refer to the pattern detected by this probe as *term/CG7271* (Fig. 4A). In Cdk9 knock-down embryos, we observed a strong reduction in *term/CG7271* expression (Fig. 4B). This phenotype could be rescued with the miRNA-resistant *Cdk9* transgene (Fig. 4B). A similar effect on *term/CG7271* expression was observed in CycT and ELL embryos, as well as in *lilli* germline clone embryos (Fig. 4B). We also measured *term*/*CG7271* expression in P-TEFb embryos by RT-qPCR, and detected reduced *term*/*CG7271* levels in both Cdk9 and CycT knock-down embryos (Fig. 3D).

To test if Term is required for embryo development, we crossed males containing a shmiRNA transgene targeting *term* with females providing maternal Gal4-VP16 to the early embryo, thereby knocking down zygotic Term expression. This shmiRNA targets a part of the 5’ UTR that is unique to *term*. Cuticle preparations from Term depleted embryos showed many embryos without any cuticle, suggesting an early arrest in development. Fixed embryos were observed with DIC microscopy, which revealed a variety of defects, including a failure to form a cellular blastoderm (Fig. 4C). In embryos homozygous for a deficiency that removes both *term* and *CG7271* as well as several other genes, we observed morphological defects to a variable extent that include a failure to form cells in part of the embryo (arrow in Fig. 4C iii). These phenotypes are not as severe as Term knock-down, indicating both on-target and off-target effects of the shmiRNA. However, the phenotypes of this deficiency are consistent with a role for *term/CG7271* in embryo development. To further analyze the function of Term, we overexpressed HA and FLAG-tagged Term ubiquitously in early embryos with maternally provided Gal4-VP16. This resulted in severe morphological defects already at the blastoderm stage (Fig. 4C iv). The *term* loss of function and gain of function morphological phenotypes appear at an earlier stage than those of P-TEFb and SEC, but support a function for Term in embryo development. Although these results do not fully explain the P-TEFb phenotypes, they indicate that changes to expression of Term in combination with other gene expression differences may mediate some of the P-TEFb and SEC morphological defects.

### Patterning genes are expressed in P-TEFb knock-down embryos

**Figure 5:**
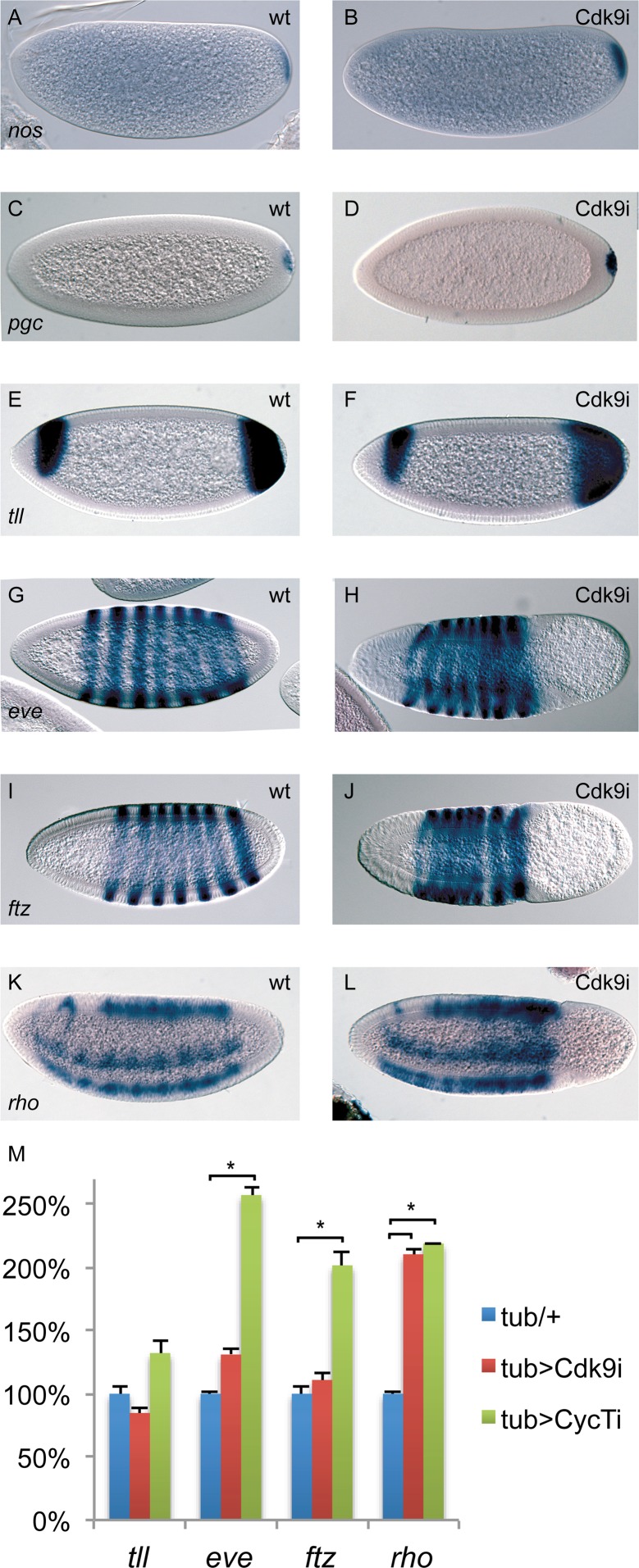
The expression of many patterning genes is relatively normal in P-TEFb embryos. Wild-type embryos (**A, C, E, G, I, K**) and embryos depleted of maternal Cdk9 (**B, D, F, H, J, L**) were hybridized with probes detecting *nanos (nos)*, *polar granule component (pgc)*, *tailless (tll)*, *even-skipped (eve)*, *fushi-tarazu (ftz)*, and *rhomboid (rho)* RNA. Relatively normal expression patterns were observed in Cdk9 embryos. (**M**) Expression of *tll*, *eve*, *ftz*, and *rho* in 2–4h P-TEFb embryos quantified by RT-qPCR (n = 4–6). Relative expression was normalized to a mean of four reference genes as described in Fig. 3. *indicates P<0.05, two-tailed paired Student’s t-test.

Many genes involved in embryo patterning are regulated by release from Pol II promoter-proximal pausing [7]. Interestingly, posterior group genes as well as genes required for development of pole cells and the embryonic termini were expressed relatively normally, despite the loss of cells from the posterior in Cdk9 embryos. As shown in Fig. 5A-D, *nanos (nos)* and *polar granule component (pgc)* mRNA is present at the posterior of pre-cellular Cdk9 knock-down embryos, indicating that the subsequent loss of cells from this part of the embryo is not due to lack of pole plasm. Similarly, *tailless (tll)* mRNA was present in the embryo posterior in cellularizing Cdk9 depleted embryos, although cells were beginning to disappear at this stage (Fig. 5E and F).

We also examined some well-studied zygotically expressed developmental genes involved in anterior-posterior and dorsal-ventral patterning. Expression of *even-skipped (eve)* and *fushi-tarazu (ftz)* occur in a 7-striped pattern in alternate parasegments in wild-type embryos (Fig. 5G and I). Although compressed by the absence of cells in the posterior, 7 stripes of *eve* and *ftz* mRNA were present in Cdk9 embryos (Fig. 5H and J). We further examined expression of *rhomboid (rho)*, which is present in two lateral stripes where it is activated by the Dorsal protein morphogen, as well as in dorsal parts of the embryo where it is expressed in response to Dpp signaling (Fig. 5K). Both the Dorsal-dependent and Dpp-dependent parts of the *rho* expression pattern were observed in Cdk9 embryos (Fig. 5L). Using RT-qPCR, we confirmed that expression of the patterning genes *tll*, *eve*, *ftz*, and *rho* were not diminished by Cdk9 or CycT knockdown (Fig. 5M). Instead, expression of *eve*, *ftz*, and *rho* were up-regulated in CycT embryos, whereas only *rho* expression was elevated in Cdk9 embryos (Fig. 5M). Up-regulation of *eve* and *ftz* in CycT, but not in Cdk9 embryos, indicates that CycT knock-down affects these genes independently of P-TEFb. Increased *rho* expression could be an indirect effect due to altered expression of *rho* regulators, or could be due to a negative function for P-TEFb at this gene. Since pausing promotes a nucleosome-free promoter region [35], one possibility is that P-TEFb depletion causes stronger pausing at *rho* that leads to a more open chromatin environment and elevated transcription. Still, the *in situ* hybridization and RT-qPCR results together suggest that expression of most genes involved in early embryo patterning is not grossly altered by reduced P-TEFb amounts.

### Diminished Pol II CTD Ser2 levels in P-TEFb knock-down embryos does not explain the gene expression phenotypes

**Figure 6:**
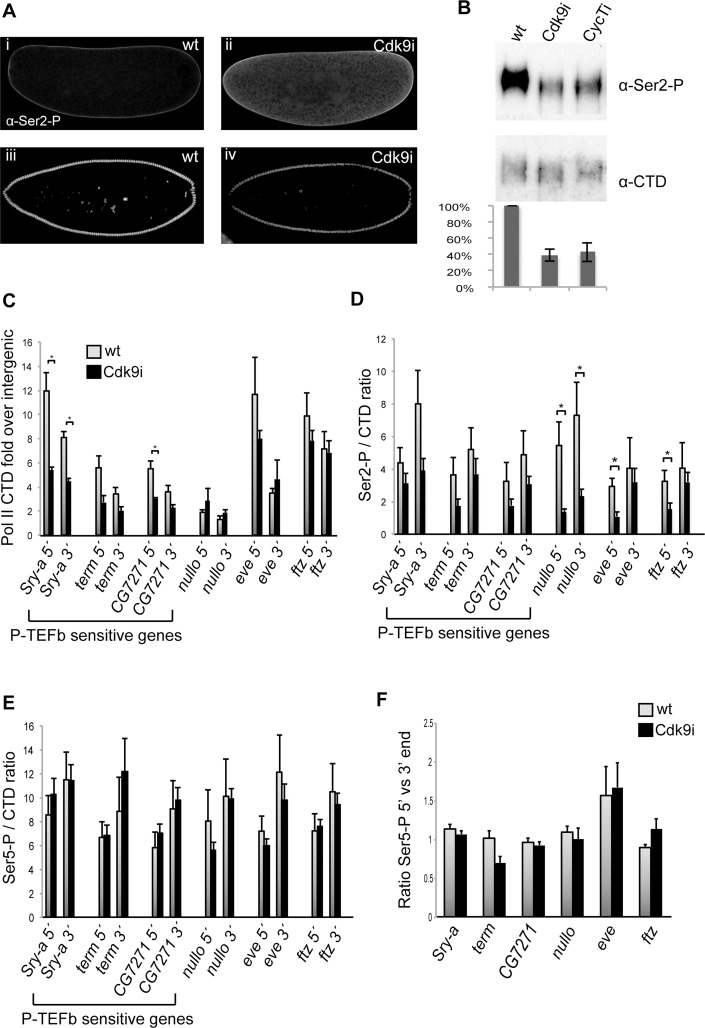
Pol II occupancy is reduced at genes affected by P-TEFb depletion. (**A**) Confocal images of wild-type embryos (i, iii) and embryos depleted of maternal Cdk9 (ii, iv) stained with an antibody recognizing phosphorylated Pol II CTD Ser2 (Ser2-P, Abcam ab5095). In pre-cellular embryos, an elevated Ser2-P signal was observed in the cytoplasm in Cdk9 embryos (ii) compared to wild-type (i), indicating that the maternal contribution of Ser2 phosphorylated Pol II was increased in Cdk9 embryos. In cellularizing embryos, less Ser2-P was detected in nuclei of Cdk9 embryos (iv) compared to wild-type nuclei (iii). (**B**) Western blot with extracts from 0–5h old embryos show a decrease in Ser2-P in embryos depleted of maternal Cdk9 or CycT. The monoclonal antibody 8WG16 recognizing the Pol II CTD was used as a loading control. The ratio of Ser2-P to CTD signal was quantified from 3 biological replicates. (**C-F**) Chromatin immunoprecipitation-quantitative PCR (ChIP-qPCR) of 2–4h wild-type or Cdk9 embryo extracts using antibodies recognizing the Pol II CTD, Pol II Ser2 phosphorylation, and Pol II Ser5 phosphorylation. (**C)** Pol II occupancy plotted as CTD enrichment relative the intergenic locus IG2c. Less Pol II associates with *Sry-α* and *CG7271* in Cdk9 embryos. (**D**) Less Ser2-P per CTD was observed in Cdk9 embryos compared to wild-type. (**E**) The Ser5-P/CTD ratio was comparable in wild-type and Cdk9 embryos. (**F**) The ratio of Pol II Ser-5 signal at the 5’ end versus the 3’ end. No increase at the 5’ end was detected in Cdk9 embryos. Error bars show standard error of the mean (n = 3–5). * indicates P<0.05, two-tailed unpaired Student´s t-test (calculations in S4 Table).

P-TEFb phosphorylates elongation factors and the Pol II CTD on Ser2 in order to release the polymerase into active elongation. We immunostained embryos to examine the localization of Ser2 phosphorylated Pol II. During the first nuclear division stages, a Pol II Ser2 signal was detected in the cytoplasm, presumably coming from polymerases not bound to chromatin (Fig. 6A). Interestingly, this signal showed an increase in Cdk9 embryos compared to wild-type. In later stage, cellularizing embryos, nuclear Pol II Ser2 was detected in all somatic cells, but not in the transcriptionally silent pole cells (Fig. 6A). Compared to wild-type embryos, reduced levels of Ser2 phosphorylated Pol II was detected in Cdk9 embryos at this stage (Fig. 6A). Quantification of the embryo stainings demonstrated that 59 +/- 1% of Ser2 phosphorylation remained in Cdk9 embryos. We further prepared extracts from embryos depleted of Cdk9 or Cyclin T and investigated global Pol II CTD Ser2 phosphorylation levels by Western blot. Embryo extracts from either Cdk9 or CycT knock-down cellularizing embryos showed lower levels of Pol II Ser2 phosphorylation (Fig. 6B), compared to the hypophosphorylated Pol IIa form of the CTD (Fig. 6B). The same results were obtained with a different Pol II Ser2 antibody (S1 Fig.). In these experiments, 39% and 43% of the phospho-Ser2 per CTD signal remained in Cdk9 and CycT embryos, respectively (Fig. 6B). Interestingly, despite different levels of Cdk9 and CycT depletion (Fig. 1), we find similar levels of Ser2 phosphorylation in Cdk9 and CycT embryos (Fig. 6B). One possibility is that the active pool of P-TEFb is depleted to the same extent in Cdk9 and CycT embryos, despite different knock-down efficiencies. Another possibility is that the activities of other Ser 2 kinases are affected by either Cdk9 or CycT depletion to the same degree. Regardless, our data show that P-TEFb knock-down during oogenesis did not reduce Pol II Ser2 phosphorylation at this stage. Instead, increased Pol II Ser 2 phosphorylation levels were found in early embryos prior to zygotic transcription. However, during cellularization, reduced amounts of Ser2 phosphorylated Pol II accumulated in the somatic nuclei of P-TEFb depleted embryos.

Next, we investigated the status of RNA Pol II at P-TEFb regulated genes by chromatin immunoprecipitation followed by quantitative PCR (ChIP-qPCR). We used antibodies against the Pol II CTD, as well as phosphorylated forms of the Pol II CTD, in extracts from 2–4h old embryos. We designed primers against the P-TEFb regulated genes *Sry-α*, *term* and *CG7271*, against *nullo*, *eve*, and *ftz* that are unchanged in Cdk9 embryos, as well as against an intergenic control locus (IG2c). We plotted CTD occupancy as fold enrichment over the intergenic region (IG2c), and observed less Pol II occupancy in Cdk9 embryos at *Sry-α* and *CG7271*, whereas no significant difference was detected at genes unaffected by Cdk9 depletion (Fig. 6C, percent input values are shown in S4 Table and in S2 Fig.). This indicates that recruitment or stability of Pol II at some genes is impaired in Cdk9 embryos.

We thereafter investigated the phosphorylation status of the polymerases associated with these genes, and normalized the amount of phosphorylation against total CTD signal. The level of Ser2 phosphorylated Pol II was decreased at all loci in Cdk9 embryos, although statistically significant differences were only obtained at insensitive genes (Fig. 6D). The result is consistent with the reduced global levels of Ser2 phosphorylation observed in Cdk9 embryos at this stage (Fig. 6A and B), but does not explain why expression of some genes were affected and others not. Ser5 phosphorylated Pol II occupancy was comparable between wild-type and Cdk9 embryos (Fig. 6E). Consistent with this finding, global Ser5 phosphorylation was unaffected in P-TEFb embryos (S3 Fig.). To measure pausing, we plotted the occupancy of Ser5 phosphorylated Pol II at the transcription start site (TSS) versus the 3’ end of the genes (Fig. 6F). Among the tested genes, we could only detect pausing for the *eve* gene, consistent with its high pausing index calculated from GRO-seq data [7]. Surprisingly, the ratio of Ser5 phosphorylated Pol II at the 5’ versus the 3’ end did not change significantly in Cdk9 embryos for any of these genes (Fig. 6F), indicating that the gene expression changes observed were not caused by increased promoter-proximal pausing. Similar results were obtained with the CTD antibody (S2 Fig.).

Taken together, our results demonstrate reduced Pol II Ser2 phosphorylation levels in cellularizing Cdk9 embryos, but this cannot explain the selective gene expression changes that occur in these embryos. Instead, Pol II occupancy is reduced at genes whose expression is diminished.

### Mediator complex subunits phenocopy P-TEFb in early embryos

**Figure 7:**
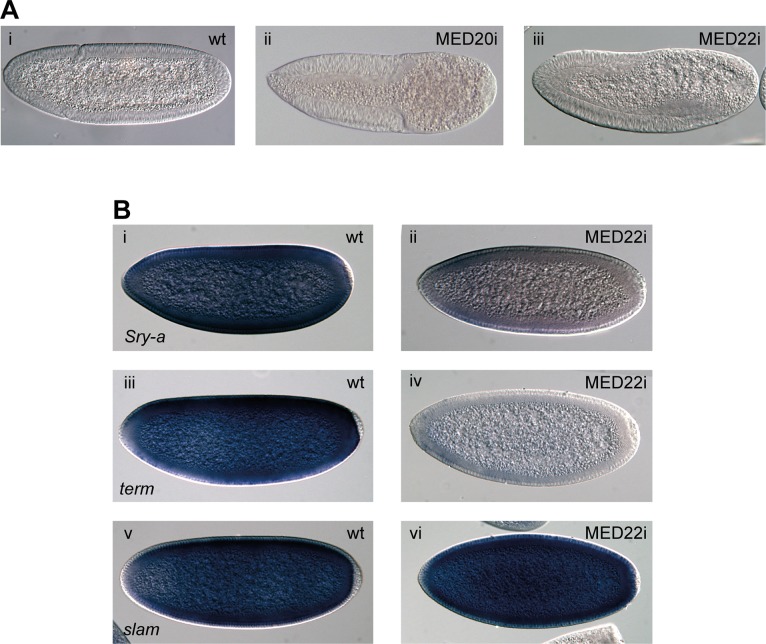
The Mediator subunits MED20 and MED22 phenocopy P-TEFb in early embryos. (**A**) DIC microscopy of cellularized wild-type (i) and maternally depleted MED20 (ii) or MED22 (iii) embryos show lack of cells in the posterior upon Mediator knock-down. (**B**) *In situ* hybridization shows that expression of *Sry-α* (ii) and *term* (iv) is compromised, whereas *slam* (vi) expression is unaffected in embryos depleted of maternal MED22. Wild-type embryos (i, iii, v) are shown for comparison.

How P-TEFb is targeted to genes is not well understood, but recent results have suggested that the Mediator subunit MED26 could be involved [17]. We therefore used shmiRNAs targeting 26 individual Mediator subunits to deplete their maternal contribution. Although MED26 knock-down did not produce an embryonic phenotype, several other Mediator subunits did (S2 Table). Both MED20 and MED22 embryos showed loss of cells from the posterior (Fig. 7A). Further analysis of MED22 embryos showed a phenotype that is virtually identical to P-TEFb embryos, including diminished *Sry-α* and *term/CG7271* expression, but no reduction in *slam* expression (Fig. 7B). These results provide *in vivo* evidence that P-TEFb and the Mediator complex cooperate in the regulation of gene expression.

## Discussion

### A subset of non-paused, pre-cellular genes are most sensitive to P-TEFb depletion

The established function of P-TEFb is to phosphorylate the RNA Pol II CTD as well as the elongation factors DSIF and NELF, allowing Pol II to enter into productive elongation [reviewed in 10]. Here, we demonstrate that embryos from which a substantial amount of the P-TEFb maternal load has been reduced show specific gene expression and morphological phenotypes. We find that some non-paused genes are more sensitive to diminished P-TEFb levels than many paused genes, consistent with recent P-TEFb inhibitor studies [11]. This provides *in vivo* evidence that also non-paused genes transit through a P-TEFb-dependent checkpoint.

P-TEFb inhibition or knock-down leads to a global decrease in Ser2 phosphorylation [28,36]. In cellularizing *Drosophila* embryos, we see a similar reduction in Ser2 phosphorylation (Fig. 6). However, the global effect on Ser2 phosphorylation does not explain the selective gene expression changes. High transcription does not explain the sensitivity to P-TEFb depletion either, since *term* and *CG7271* are expressed at similar levels to *slam* and *bnk* (S4 Fig. and [7]). Instead, the P-TEFb down-regulated genes that we have identified are non-paused, which would suggest that they require P-TEFb continuously for efficient release into elongation. However, the state of pausing is not the only determinant for sensitivity to P-TEFb depletion since *bnk*, *slam* and *nullo* are also non-paused, rapidly and highly induced in pre-cellular embryos and regulated by the transcription factor Zelda, but not affected by P-TEFb knock-down. Moreover, it is possible that among the many paused genes in the early embryo, some that are also down-regulated by P-TEFb depletion could be detected by further investigation. Moreover, although both *rho* and *eve* are highly paused genes in the embryo [7], expression of *rho*, but not *eve*, is increased upon Cdk9 knock-down. Therefore, as yet unidentified features of P-TEFb-regulated genes confer sensitivity to reduced P-TEFb levels.

Surprisingly, the ratio of Pol II at the promoter versus the 3’ end at P-TEFb down-regulated genes does not change in Cdk9 embryos (Fig. 6F). Since no difference in Pol II distribution along the tested genes could be noted, Pol II appears to be released into elongation to the same extent in P-TEFb and wild-type embryos. Rather, it appears that compared to unaffected genes, less Pol II associates with genes whose expression is reduced in P-TEFb embryos (Fig. 6C). Thus, lowered Pol II occupancy may explain diminished transcription of some genes in P-TEFb embryos. Consistent with this idea, analysis of global run-on sequencing (Gro-seq) data suggests that the *Sry-α* and *CG7271* genes are regulated at the Pol II recruitment step, and not by release from pausing in early embryos [7]. Furthermore, Pol II ChIP-seq has shown that none of the P-TEFb down-regulated genes ever display pausing during development [25].

Why is there less Pol II associated with some genes in P-TEFb embryos? It is possible that P-TEFb regulates expression of transcription factors during oogenesis that are needed for Pol II recruitment to P-TEFb-regulated genes. One such candidate is Zelda, which is required for zygotic genome activation [23]. Since *zelda* embryos show phenotypes similar to P-TEFb and also regulates the P-TEFb-sensitive genes *Sry-α*, *term*, and *CG7271* [23], we tested if maternal transcript levels of *zelda* are affected in P-TEFb embryos. *In situ hybridization* of P-TEFb depleted embryos showed that *zelda* mRNA levels are comparable to wild type embryos (S5 Fig.), suggesting that P-TEFb is not controlling maternal expression of *zelda*. P-TEFb might control transcription of other maternal factors that play a role in Pol II recruitment to P-TEFb-sensitive genes. Another possibility is that P-TEFb has a more direct function in recruiting Pol II to a specific set of promoters. This alternate function of P-TEFb and SEC could be evolutionarily conserved, since knocking down the SEC component ELL2 in mouse embryonic stem cells affects Pol II occupancy at the non-paused *Cyp26a1* gene [16]. A recent study showed that whereas a majority of genes in mouse embryonic stem cells accumulate Pol II at the promoter after P-TEFb inhibition, around 20% showed a decrease in Pol II promoter occupancy [11]. Yet one more possibility is that cross talk between pausing and initiation explains why these genes and P-TEFb down-regulated genes in the *Drosophila* embryo have reduced Pol II occupancy. Inhibiting release into elongation may feed back on transcription initiation and result in decreased Pol II levels.

### P-TEFb, SEC and Mediator share phenotypes in the *Drosophila* embryo

A fraction of P-TEFb is present in the Super Elongation Complex (SEC). The SEC components ELL and Lilliputian (dAFF4) have previously been shown to be required for embryo development and segmentation [24,37]. We demonstrate that P-TEFb and these SEC components display similar morphological and gene expression phenotypes in early embryos (Figs. 3 and 4), providing *in vivo* evidence that P-TEFb function is mediated at least in part as a component of the SEC. However, *lilli* mutant embryos are different in some respects from P-TEFb knock-down embryos. Expression of some genes, including *ftz*, is reduced in *lilli* mutant embryos [24], but not by P-TEFb depletion (Fig. 5). This could be because this *lilli* allele is a stronger loss of function mutation that reduces Lilli protein levels more than P-TEFb is reduced by the shmiRNAs. Alternatively, this SEC component has functions that do not require P-TEFb. Importantly, many other chromatin and transcriptional regulators that we knocked-down using shmiRNAs did not share embryonic phenotypes with P-TEFb and SEC components, demonstrating the specificity of the phenotype and allowing for the identification of factors that contribute to P-TEFb and SEC function in the embryo.

The Mediator complex was purified based on its ability to mediate activated transcription by bridging upstream transcription factors with Pol II, but additional functions for Mediator have emerged recently [reviewed in 38]. The Mediator subunit MED26 interacts with Eaf, a member of the SEC, and recruits elongation factors to promoters in mammalian cells [17]. It has also been shown that mammalian MED23 can recruit P-TEFb by interacting with Cdk9 [39], and that HIF1a can recruit the SEC via the CDK8 Mediator subunit in response to hypoxia [40]. We find that depletion of several *Drosophila* Mediator subunits phenocopy P-TEFb embryos and result in identical gene expression changes (Fig. 7). Our results are consistent with a Mediator-SEC interaction that is important for gene transcription *in vivo*, and indicate that Mediator and SEC function together not only to control elongation, but also in recruiting Pol II to some developmental genes.

### P-TEFb and germ cells

In many organisms, germ cells are specified early during embryo development. In order to prevent these cells from differentiating into somatic cells, mRNA expression is transiently, but globally, repressed [reviewed in 41]. A common strategy to specifically prevent Pol II transcription has evolved that involves inhibition of Ser2 phosphorylation [41]. In *Drosophila*, *polar granule component* (*pgc*) is the germ plasm factor that represses Pol II transcription and Ser2 phoshorylation in the pole cells [42,43], by preventing P-TEFb from associating with chromatin [42]. We find that *pgc* is expressed and that pole cells are generated in P-TEFb embryos (Fig. 5), despite loss of cells from the embryo posterior at later stages (Fig. 2).

In *C*. *elegans*, the PIE-1 protein binds to CycT and prevents Ser2 phosphorylation in the germline blastomeres [44,45,46]. In contrast to somatic cells, loss of Cdk9 from mature germ cells has little effect on Ser2 phosphorylation, whereas Cdk12 loss abolishes Ser2 phosphorylation [47]. Interestingly, Cdk12 and Ser2 phosphorylation are not required for *C*. *elegans* germline development and function, whereas Cdk9 is essential [47]. Thus, P-TEFb has substrates other than the Pol II CTD that are needed for *C*. *elegans* germline function.

Interestingly, we detect elevated levels of Pol II Ser 2 phosphorylation in pre-cellular P-TEFb embryos (Fig. 6), indicating that P-TEFb is not responsible for Ser2 phosphorylation in the *Drosophila* female germline. We used shmiRNAs targeting Cdk12 and CycK in the germline, but these females failed to produce eggs, demonstrating that Cdk12 is required for oogenesis. Thus, there are both similarities and differences between the *C*. *elegans* and *Drosophila* germline, but in both organisms P-TEFb appears to function differently in germ cells and somatic cells.

### Terminus is essential for embryo development

Staining of nuclei and the plasma membrane in P-TEFb embryos demonstrated cellularization defects, including multinucleated cells, and showed that cells are also lost from the posterior after cellularization (Fig. 2). Damaged nuclei or nuclei with cell cycle defects can trigger a similar phenotype, nuclear fallout, thereby preventing them from becoming somatic nuclei [48]. It is possible that P-TEFb depletion causes cell cycle perturbations that result in the observed phenotype, although no obvious chromosome segregation defects were detected in the embryo posterior. Another possibility is that gene expression is perturbed in the embryo posterior by P-TEFb depletion, causing the nuclei to detach from the cortex.

A rather small number of zygotically transcribed genes are known to control cellularization. We have identified an additional Zelda, SEC, and P-TEFb regulated gene that could be involved in this process. The gene *terminus* (*term*) was identified based on its blastoderm-specific expression [34]. Expression becomes restricted to the posterior in cellularized embryos, at the same time as we observe cell loss in the embryo posterior in P-TEFb embryos. We discovered that shmiRNA knockdown of Term or deletion of a large genomic region that includes Term is lethal to embryos and results in morphological defects, including a failure to form a cellular blastoderm (Fig. 4). Over-expression of Term similarly causes morphological deformations of early embryos (Fig. 4). These results show that Term is essential for early development, and indicate that Term may play a role in cellularization. However, the *term* phenotypes are different from the lack of cells observed in the posterior of cellularized P-TEFb embryos. We therefore favor the idea that the P-TEFb phenotype is caused by multiple gene expression changes.

The molecular function of Term is unexplored. It encodes a 428 amino acid (aa) protein with a single C2H2-type zinc-finger. Term is closely related to CG7271, 423 out of the 428 aa are identical. Term also shows 28% aa homology to the CG6885 gene product, in which the zinc-finger is also conserved. These three genes have very similar gene expression profiles with transcription restricted to early zygotic activation (Flybase RNA-seq data, [33]). However, none of these genes are conserved outside the *Drosophila* genus, suggesting that species within this clade have adopted them to perform an essential early embryonic function.

### P-TEFb depleted embryos express promoter-proximal paused patterning genes

A large fraction of the genes involved in early embryo patterning, both the ones controlling anterior-posterior development and those involved in dorsal-ventral patterning, contain a promoter-proximal paused Pol II. Recent Gro-seq experiments have indicated that the majority of these are regulated by release from pausing [7]. Given the function of P-TEFb in releasing Pol II from pausing into active elongation, it could be expected that these paused genes would be susceptible to reduced P-TEFb amounts. Indeed, mitotic *Cdk9* clones in imaginal discs demonstrated various patterning defects and reduced expression of Hox genes that contain paused Pol II [29]. However, inhibiting P-TEFb activity with flavopiridol showed that highly paused genes are less susceptible to P-TEFb inhibition than non-paused genes [11], indicating that they experience release from pausing less frequently than non-paused genes. We found that the majority of segmentation and dorsal-ventral genes are expressed in a relatively normal embryonic pattern, despite the morphological changes and loss of cells from P-TEFb knock-down embryos. Instead, we found that some non-paused genes are most sensitive to reduced P-TEFb levels.

Our results are consistent with a model for metazoan gene transcription where all genes require P-TEFb-mediated escape from pausing, and where non-paused genes rely most heavily on rapid release into elongation. In this model, non-paused genes will be most sensitive to diminished P-TEFb levels. Our results are also in line with studies of the SEC in mammalian cells, which showed that P-TEFb and SEC components are enriched on highly transcribed genes that are rapidly induced [16]. SEC depletion resulted in decreased Pol II occupancy of both paused and non-paused genes [16]. Although P-TEFb and the SEC may directly regulate Pol II recruitment to rapidly transcribed genes in conjunction with the Mediator complex, P-TEFb-regulated release from pausing could also feedback on transcription initiation.

## Materials and Methods

Primer sequences are listed in S3 Table.

### Molecular cloning, transgenic flies and crosses

The *Cdk9* gene region was PCR amplified with primers containing an XbaI overhang cdk9GR2_f and cdk9GR2_r that bind 410 bp upstream of the *Cdk9* TSS and 970 bp downstream of the 3´ UTR. The PCR product was cloned into the pAttB vector [49], resulting in the plasmid pAttB-Cdk9. A TY1-V5-FLAG tag was attached to the C-terminus of *Cdk9* directly upstream of the stop codon by *in vivo* recombineering. A selection cassette having TY1-V5-FRT-KanR-RpsL-FRT-FLAG was PCR amplified from the pTAGNG vector [50], (kindly provided by Mihail Sarov) using the primers cdk92TY_f + cdk9flag_R with overhangs containing homology to the *Cdk9* C-terminal region. *In vivo* recombineering of the tag into pAttB-Cdk9 was carried out by growing the pAttB-Cdk9 plasmid in a HME71 bacterial strain [51], kindly provided by the Donald Court lab. Cells were heat shocked to induce Red recombineering proteins followed by electroporation of the PCR amplified tagging cassette into the cells. Colonies were selected for Kanamycin-resistance, and verification of correctly recombineered pAttB-Cdk9 vector performed by DNA sequencing. The Cdk9 construct was then electroporated into 10-beta *E*. *coli* cells together with the pSC101-BAD-Flpe-tet plasmid provided by Francis Stewart. The KanR-RpsL sequence in the tagged pAttB-Cdk9 vector was flipped out by inducing FLP expression with 0.2% L-arabinose. Clones were selected for streptomycin resistance confirming the loss of the KanR-RpsL sequence. This left only the TY1-V5-FLAG-tag and an FRT scar in frame in the C-terminus of *Cdk9*, which was verified by sequencing.

A miRNA resistant sequence was introduced into pattB-Cdk9 by PCR amplification of the entire vector using two phosphorylated primers, cdk9GR3_f + cdk9GR3_r, both having overhangs containing one half of the miRNA resistant sequence each. The PCR reaction was Dpn I-treated and blunt-ligated to circularize the PCR product into a plasmid. Correct clones were identified by restriction digestion and DNA sequencing.

All pAttB-Cdk9 transgenic constructs were introduced into the attP40 landing site on the second chromosome, and combined with the TRIP shmiRNA lines on chromosome 3. In order to verify the existence of the *Cdk9* shmiRNA on the third chromosome after combination with the miRNA-resistant transgene, we performed PCR with valium20_f + valium20_r primers.

The shmiRNA knockdown strains (created by the Harvard TRiP project) used were:

***Cdk9*:** *y* ^*1*^ sc* v ^*1*^; P{y[+t7.7] v[+t1.8] = TRiP.HMS01391}attP2 (hairpin ID SH01793.N) ***CycT*:** *y* ^*1*^ sc* v ^*1*^; P{y[+t7.7] v[+t1.8] = TRiP.HMS00776}attP2 (hairpin ID SH00839.N) ***dEll*:** *y* ^*1*^ sc* v ^*1*^; P{y[+t7.7] v[+t1.8] = TRiP.HMS00277}attP2 (hairpin ID SH00582.N), ***term*:** *y* ^*1*^ sc* v ^*1*^; P{y[+t7.7] v[+t1.8] = TRiP.GLV21046}attP2 (hairpin ID SH01121.VP)

***Cdk12*:** *y* ^*1*^ sc* v ^*1*^; P{y[+t7.7] v[+t1.8] = TRiP.HMS00155}attP2 (hairpin ID SH00274.N)

***CycK*:** *y* ^*1*^ sc* v ^*1*^; P{y[+t7.7] v[+t1.8] = TRiP.HMS01003}attP2 (hairpin ID SH01772.N)

***MED20*:** *y* ^*1*^ sc* v ^*1*^; P{y[+t7.7] v[+t1.8] = TRiP.HMS01051}attP2 (hairpin ID SH01723.N)

***MED22*:** *y* ^*1*^ sc* v ^*1*^; P{y[+t7.7] v[+t1.8] = TRiP.HMS01047}attP2 (hairpin ID SH01717.N)

Additional Mediator subunit hairpin IDs are listed in S2 Table.

The w*; P{w[+mC] = matalpha4-GAL-VP16}V2H strain was used as maternal Gal4 driver. pUAS-*Term*-HA-FLAG was a kind gift from Krishnaswamy VijayRaghavan, the DPIM project. The w*; P{w[+mC] = matalpha4-GAL-VP16}V2H driver was combined with w*; P{w[+mC] = His2Av-mRFP1}III.1, made homozygous for both transgenes and used for live imaging. Females containing *lilli* germ-line clones were generated as previously described [52], using the stocks *lilli* ^*XS575*^ *FRT40A/CyO*, *hsFLP w* ^*1118*^ *; Adv* ^*1*^ */CyO* and *ovoD* ^*1*^ *FRT40A/Dp(*?*;2)bw* ^*D*^, *S* ^*1*^ *wg* ^*Sp-1*^ *Ms(2)M* ^*1*^ *bw* ^*D*^ */CyO*. Embryos were collected from the *w* ^*1118*^ *; Df(3L)BSC416/TM6C*, *Sb* stock, which contains a deficiency that removes *term*, *CG7271*, *CG6885*, and around 50 additional genes.

### Collection of knock-down embryos

The w*; P{w[+mC] = matalpha4-GAL-VP16}V2H strain was used as maternal Gal4 driver. At least 50 virgin homozygous TubGal4 females were placed in food bottles together with males carrying the miRNA transgene of interest. The progeny, F1 females and males, were allowed to mate with each other in the food bottles for 2–4 days before being transferred to embryo collection cages. Embryos were collected from the cages on fruit juice agar plates supplemented with yeast.

### miRNA resistant rescue assay

Overnight embryos were handpicked and placed on yeast-covered juice agar plates and put in standard fly food bottles. Eclosed live flies as well as dead flies stuck in the food were counted as rescued.

### *In situ* hybridization

The whole-mount embryo RNA *in situ hybridization* protocol was modified from the protocol used in [53,54]. Probes against *slam*, *bnk*, *nullo* and *Sry-α* were generated by PCR from genomic DNA, A-overhangs added with Taq-polymerase and TA-cloned into pGEM-T Easy (Promega). Anti-sense DIG-UTP labeled RNA probes were made with SP6 or T7 RNA polymerase. For *term/CG7271*, *pgc* and *zelda*, an SP6 overhang was directly added to the PCR primer, and the PCR product was used as a template for in vitro transcription. Probes for *eve*, *ftz*, *tll*, *nos*, and *rho* were generated previously.

### Quantitative RT-PCR

Total RNA was extracted from embryos using Trizol (Invitrogen) according to the manufacturer´s protocol. For one biological replicate 20 ul embryos were collected. A total of 1 ug from each RNA sample was subjected to DNase digestion (Sigma) and cDNA synthesis (Applied Biosystems) according to the manufacturer´s protocol. Samples were confirmed for quality and quantity using agarose gel electorphoresis and Nanodrop. Out of 200 μl (10x diluted) total cDNA, 2 μl was subsequently used for RT-qPCR in a total volume of 20 μl using CFX96 (BioRad instruments, Sweden) with EvaGreen supermix (Solis BioDyne). Resulting Ct-values were converted into raw quantities with the ΔCt method using Genorm, which were used to calculate the geometric mean of four reference genes ß-tubulin, RpL32 28SrRNA and GAPDH, serving as normalization factors for each cDNA sample. The stability measure M [55], for the mean of the reference genes was below 1.5 for all the conditions tested. Relative expression levels were calculated where expression in control embryos derived from mothers with only the TubGal4 transgene was set to 100%.

### Immunoflourescence and live imaging

Embryos were collected and fixed as for RNA *in situ hybridization* and stained in PBT buffer using *α*-phosphotyrosine (1:150, 4G10, Millipore 05–321), *α*-H4 (1:400, Abcam ab10158) or *α*-Pol II Ser2 (1:200, Abcam ab5095). Cy3 and Alexa-488 flourescent secondary antibodies (Invitrogen) were used at a 1:400 dilution. Embryos were visualized in either a Zeiss LSM 780 or a Zeiss LSM 510 Meta confocal microscope, and images acquired and adjusted with Zen software (Zeiss).

For widefield live imaging, a fly strain carrying the Tubulin-Gal4-VP16 transgene and a Histone 2Av-RFP fusion transgene was created. The RFP flies were then crossed with the Cdk9 miRNA strain or used directly as a wild-type control. Embryos were collected for 30–60 min and dechorionated in bleach, then aligned on a gas-permeable membrane on top of two silicone bars and covered in halocarbon oil. Embryos were imaged every 2 min on a Zeiss AxioImagerZ1 microscope equipped with a Zeiss plan-apochromat 20X/0.75NA objective. Imaging was controlled by AxioVison software. Images were assembled with ImageJ software into time-lapse movies. Contrast and brightness settings were applied to the movies with ImageJ for better visualization.

### Western blot

Protein extracts were resolved on SDS-PAGE, transferred to PVDF membrane, and incubated with primary antibodies diluted in PBS with 2% BSA: *α*-Histone3 (1:5000, Abcam ab1791), *α*-CyclinT (1:1000, gift from David Price), *α*-FLAG (1:2000, M2, Sigma F3165), *α*-CTD (1:500, 8WG16, Abcam ab817), *α*-Ser2 (1:500, Abcam ab5095), *α*-Ser2 (1:1000, clone 3E10, Millipore 04–1571), *α*-Ser5 (1:1000, Abcam ab5131), rat *α*-HDAC3 (1:1000, [56]) or *α*-alpha-Tubulin (1:2000, Abcam ab18251). HRP-labeled secondary antibodies (1:10 000 in PBS with 3% milk) were detected with ECL Select reagent (GE Healthcare) and the membrane exposed to a Luminescent Image Analyzer (LAS-100plus, Fujifilm). Quantification used Fuji Gauge Image software, and CycT signal was compared to HDAC3 loading control, whereas Cdk9-FLAG signal was compared to histone H3.

### Chromatin immunoprecipitation

Chromatin IP was performed as described in [57], and 3–5 independent biological replicates were produced for each antibody. A dounce homogenizer was used to grind 2–4h old wild-type or Cdk9 *Drosophila* embryos and a Diagenode Bioruptor was used to sonicate the material before IP. The antibodies used were *α*-Pol II CTD (8 μl, 8WG16, Abcam ab817), *α*-Pol II Ser2 (7 μl, Abcam ab5095), *α*-Ser5 (5 μl, Abcam ab5131). Twenty-five or 30 μl Protein A/G-coated magnetic bead mix slurry and 40–50 μl of embryos were used for each IP. ChIP samples were eluted in 160 μl 0.1x TE pH 8 and duplicates with 2 μl DNA each analyzed by quantitative PCR using a Bio-Rad CFX96 machine and HOT FIREPol EvaGreen master mix (Solis BioDyne). From each IP, the percent of input signal was obtained using the average of the two duplicates, and the Ser5/CTD and Ser2/CTD ratios calculated using these values. Pol II occupancy and Pol II 5’/3’ ratios were calculated by normalizing CTD percent input signal against an intergenic locus. Student´s unpaired t-test for two-sample equal variance was used to find statistically significant differences (p<0.05) between control and Cdk9 samples. All values and calculations can be found in S4 Table.

## Supporting Information

S1 Fig Pol II Ser2 phosphorylation in Cdk9 embryos. Immunostaining and Western blot using the anti-Ser2 phosphorylation antibody 3E10 (Millipore). (**A**) Cellularizing wild-type embryos (i) and embryos depleted of maternal Cdk9 (ii) were stained with the 3E10 antibody. (**B**) Western blot of 0–30 min old wild-type embryos and embryos depleted of maternal Cdk9 probed with phospho-Ser2 (3E10), CTD (8WG16), and tubulin antibodies. Increased Ser2 phoshorylation was detected in Cdk9 embryos at this stage. (**C**) In extracts derived mainly from cellularizing and cellularized embryos (0–5 h), less Ser2 phosphorylation (a-Ser2p, 3E10) was observed compared to the CTD (8WG16) loading control.
(PDF)

S2 Fig ChIP-qPCR with Pol II antibodies in Cdk9 embryos. ChIP-qPCR of 2–4h wild-type or Cdk9 embryo extracts using antibodies recognizing the Pol II CTD, Pol II Ser2 phosphorylation (Ser2-P), and Pol II Ser5 phosphorylation (Ser5-P) plotted as percent of input. (**A)** Pol II CTD enrichment. (**B**) The ratio of Pol II CTD at the 5’ end versus the 3’ end. No increased CTD signal at the 5’ end was detected in Cdk9 embryos. (**C**) Ser2-P enrichment. (**D**) Ser5-P enrichment. Error bars show standard error of the mean (n = 3–5).
(PDF)

S3 Fig Global Pol II Ser5 phosphorylation levels are not changed in P-TEFb embryos. Western blot with extracts from 2–4h old embryos show similar levels of Ser5-P in embryos depleted of maternal Cdk9 or CycT as in control embryos derived from mothers with only the TubGal4 transgene. The 8WG16 antibody that recognizes the Pol II CTD was used as a loading control and used to calculate a Ser5-P/CTD ratio. A lane with twice the volume was also loaded for each sample.
(PDF)

S4 Fig Relative gene expression in control embryos. RT-qPCR was used to relate expression of the zygotic genes assayed in ChIP experiments (Fig. 6) in control embryos (derived from mothers with the TubGal4 transgene) relative the housekeeping gene *Rp49* (*RpL32*, which is maternally contributed). Columns show average values in 2–4h embryos with S.E.M. (n = 6), and *Rp49* expression was set to 1. Note the logarithmic scale.
(PDF)

S5 Fig Expression of *zelda* in Cdk9 embryos. Pre-cellular wild-type (**A**) and maternal Cdk9-depleted (**B**) embryos hybridized with a digoxigenin-labeled *zelda* probe. No difference in the maternal contribution of *zelda* mRNA was detected.
(PDF)

S1 Table Rescue of lethality by the miRNA-resistant Cdk9 transgene. Embryos were collected from mothers depleted of Cdk9 in the germline or from Cdk9-depleted mothers that also had a *Cdk9* miRNA-resistant transgene, and the number of offspring that survived to adulthood was counted.
(PDF)

S2 Table Embryonic phenotypes of Mediator subunits. The maternal contribution of 26 individual Mediator subunits was knocked-down. Embryos were collected from females containing the maternal α-Tubulin-Gal4-VP16 driver and shmiRNAs targeting Mediator components, and cuticle preparations examined by dark-field microscopy.
(PDF)

S3 Table Primer sequences. (XLSX)

S4 Table ChIP-qPCR raw data. ChIP from wild-type and Cdk9 2–4 h old embryo extracts with antibodies against the Pol II CTD, phospho-Ser5, and phosho-Ser2. Percent input values of individual replicates, and the calculations and statistics used for Fig. 6 are shown.
(XLS)

S1 Movie Live imaging of H2Av-RFP labeled nuclei in wild-type embryos. Embryos derived from mothers with a histone H2Av-RFP transgene and the TubGal4 driver (Movie S1, wild-type control) were dechorionated and imaged every 2 min. Images were assembled with ImageJ software into time-lapse movies.
(MP4)

S2 Movie Live imaging of H2Av-RFP labeled nuclei in Cdk9 depleted embryos. Embryos derived from mothers with a histone H2Av-RFP transgene, TubGal4, and Cdk9 shmiRNA (Movie S2, Cdk9i) were dechorionated and imaged every 2 min. Images were assembled with ImageJ software into time-lapse movies. The nuclear fallout in the center of the Cdk9 embryo may be caused by the presence of the H2Av-RFP transgene, as it was also often observed in control embryos, whereas the loss of cells from the embryo posterior was specific to Cdk9 embryos.
(MP4)
