# Supplementary figures and images for: P-TEFb, the Super Elongation Complex and Mediator Regulate a Subset of Non-paused Genes during Early Drosophila Embryo Development
Source: PLoS Genet. 2015 Feb 13;11(2):e1004971. doi: 10.1371/journal.pgen.1004971 (PMC4334199; doi:10.1371/journal.pgen.1004971)

**A**

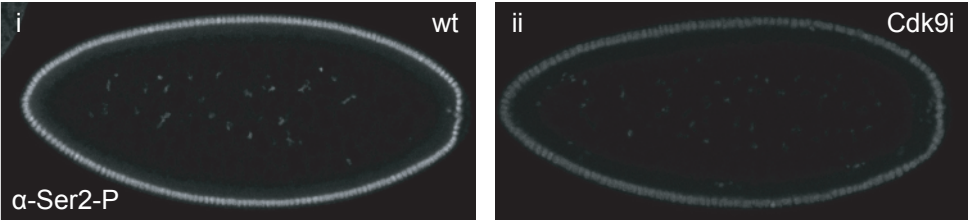

**B**

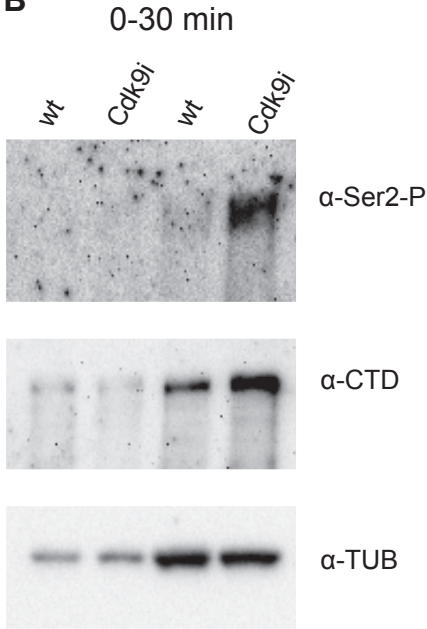

**C**

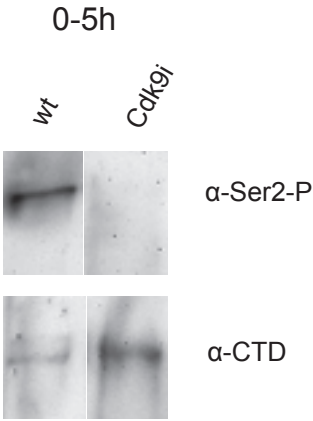

Supplement: S1 Fig — Immunostaining and Western blot using the anti-Ser2 phosphorylation antibody 3E10 (Millipore). (A) Cellularizing wild-type embryos (i) and embryos depleted of maternal Cdk9 (ii) were stained with the 3E10 antibody. (B) Western blot of 0–30 min old wild-type embryos and embryos depleted of maternal Cdk9 probed with phospho-Ser2 (3E10), CTD (8WG16), and tubulin antibodies. Increased Ser2 phoshorylation was detected in Cdk9 embryos at this stage. (C) In extracts derived mainly from cellularizing and cellularized embryos (0–5 h), less Ser2 phosphorylation (a-Ser2p, 3E10) was observed compared to the CTD (8WG16) loading control. (PDF) [file pgen.1004971.s001.pdf]

**A**

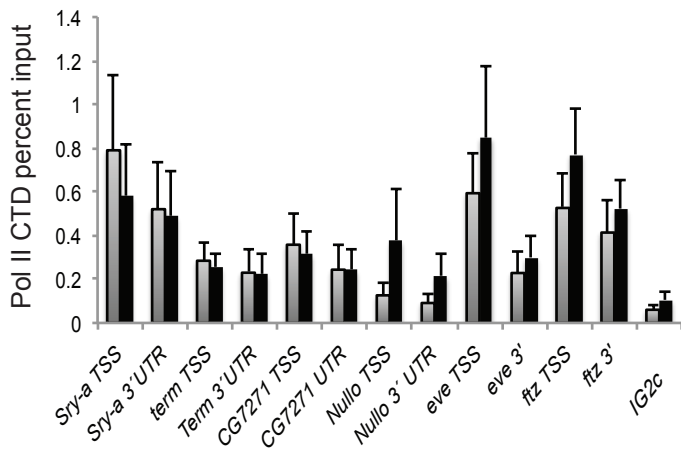

**C**

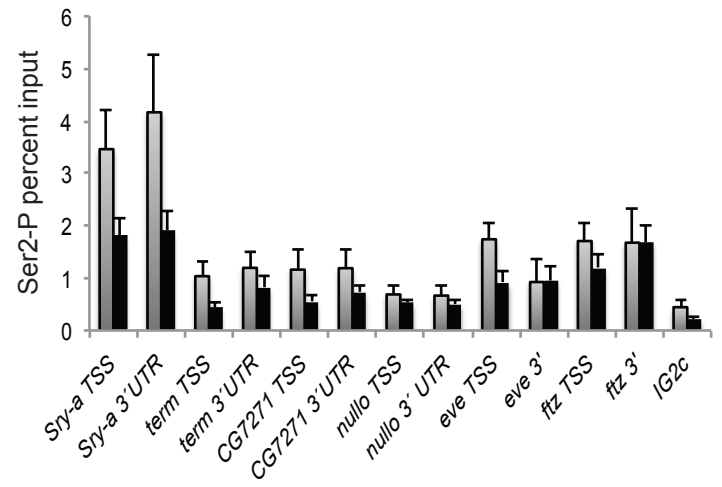

**B**

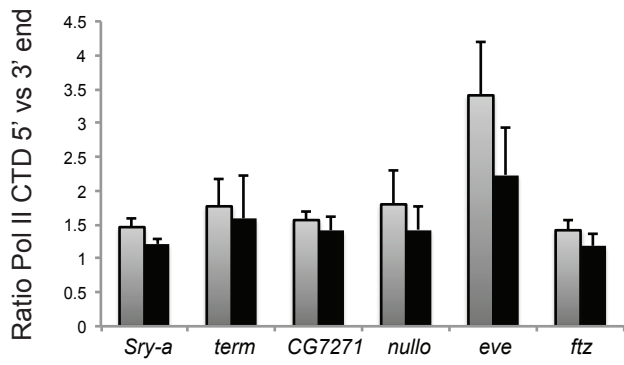

**D**

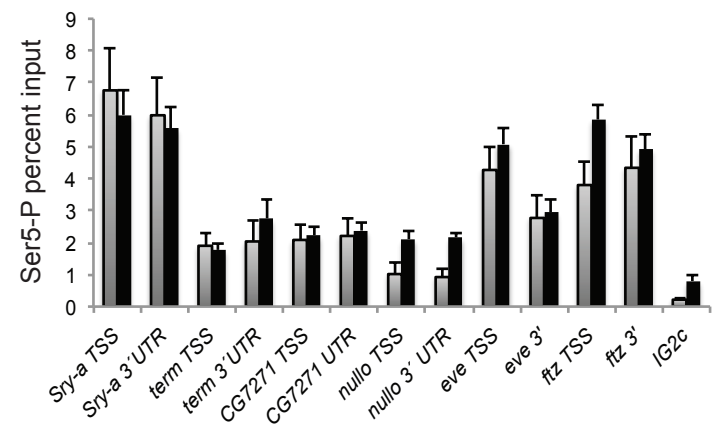

Supplement: S2 Fig — ChIP-qPCR of 2–4h wild-type or Cdk9 embryo extracts using antibodies recognizing the Pol II CTD, Pol II Ser2 phosphorylation (Ser2-P), and Pol II Ser5 phosphorylation (Ser5-P) plotted as percent of input. (A) Pol II CTD enrichment. (B) The ratio of Pol II CTD at the 5’ end versus the 3’ end. No increased CTD signal at the 5’ end was detected in Cdk9 embryos. (C) Ser2-P enrichment. (D) Ser5-P enrichment. Error bars show standard error of the mean (n = 3–5). (PDF) [file pgen.1004971.s002.pdf]

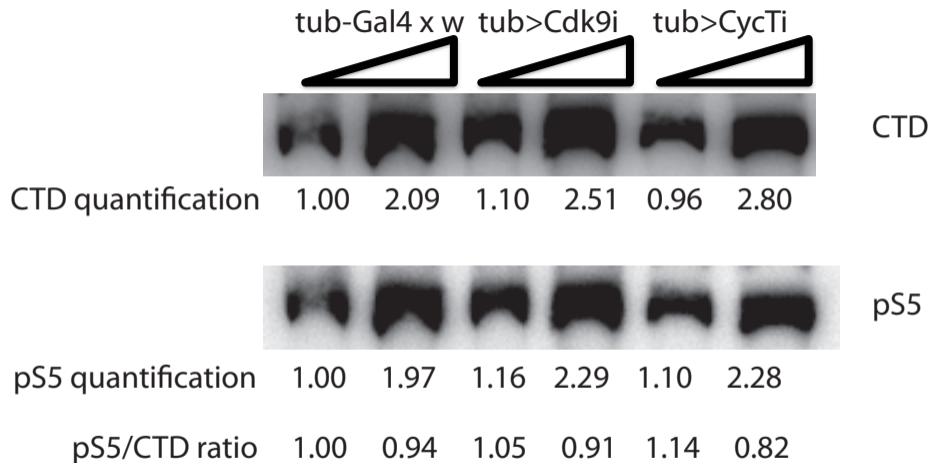

Supplement: S3 Fig — Western blot with extracts from 2–4h old embryos show similar levels of Ser5-P in embryos depleted of maternal Cdk9 or CycT as in control embryos derived from mothers with only the TubGal4 transgene. The 8WG16 antibody that recognizes the Pol II CTD was used as a loading control and used to calculate a Ser5-P/CTD ratio. A lane with twice the volume was also loaded for each sample. (PDF) [file pgen.1004971.s003.pdf]

Dahlberg Fig. S4

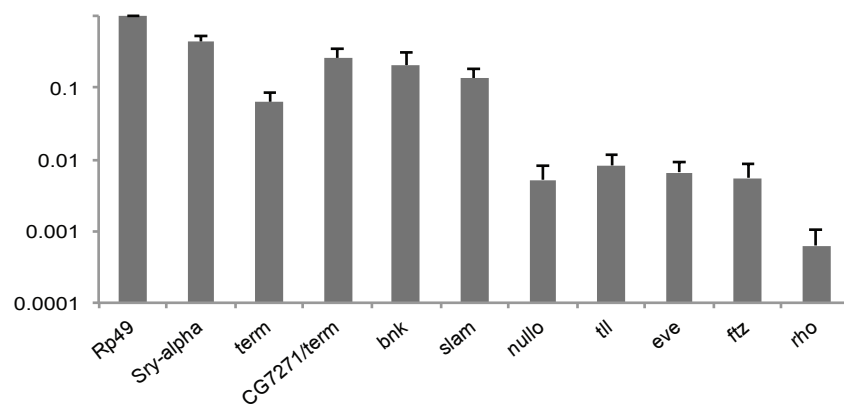

Supplement: S4 Fig — RT-qPCR was used to relate expression of the zygotic genes assayed in ChIP experiments (Fig. 6) in control embryos (derived from mothers with the TubGal4 transgene) relative the housekeeping gene Rp49 (RpL32, which is maternally contributed). Columns show average values in 2–4h embryos with S.E.M. (n = 6), and Rp49 expression was set to 1. Note the logarithmic scale. (PDF) [file pgen.1004971.s004.pdf]

Dahlberg Fig. S5

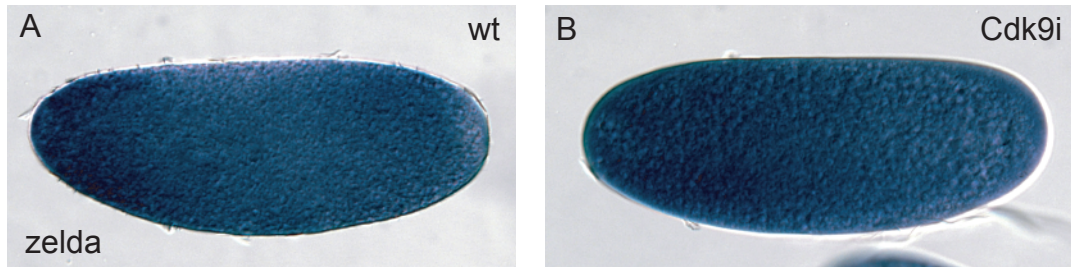

Supplement: S5 Fig — Pre-cellular wild-type (A) and maternal Cdk9-depleted (B) embryos hybridized with a digoxigenin-labeled zelda probe. No difference in the maternal contribution of zelda mRNA was detected. (PDF) [file pgen.1004971.s005.pdf]
