# Supplementary material for: P-TEFb, the Super Elongation Complex and Mediator Regulate a Subset of Non-paused Genes during Early Drosophila Embryo Development
Source: PLoS Genet. 2015 Feb 13;11(2):e1004971. doi: 10.1371/journal.pgen.1004971 (PMC4334199; doi:10.1371/journal.pgen.1004971)
Supplement: S1 Table — Embryos were collected from mothers depleted of Cdk9 in the germline or from Cdk9-depleted mothers that also had a Cdk9 miRNA-resistant transgene, and the number of offspring that survived to adulthood was counted. (PDF) [file pgen.1004971.s006.pdf]

Parental genotype (♀ x ♂)	Number of adult offspring
**Maternal Cdk9 depletion (TubGal4/+; shmiRNA Cdk9/+)**	0/163 (0%)
**Maternal Cdk9 depletion; miRNA resistant Cdk9 transgene (TubGal4/Cdk9 resistant; shmiRNA Cdk9/+)**	109/151 (72%)
