# Supplementary material for: P-TEFb, the Super Elongation Complex and Mediator Regulate a Subset of Non-paused Genes during Early Drosophila Embryo Development
Source: PLoS Genet. 2015 Feb 13;11(2):e1004971. doi: 10.1371/journal.pgen.1004971 (PMC4334199; doi:10.1371/journal.pgen.1004971)

Gal4 Driver	Gene symbol	Annotation	Hairpin ID	Cuticle phenotype
TubGal4	MED1	CG7162	SH01794.N	**wt phenotype**
TubGal4	MED4	CG8609	SH01718.N	**no F1 flies hatched**
TubGal4	MED6	CG9473	SH01719.N	**100% undeveloped embryos, very few eggs laid**
TubGal4	MED7	CG31390	SH01795.N	**100% penetrance**
TubGal4	MED8	CG13867	SH01784.N	**no eggs laid**
TubGal4	MED9	CG42517	SH00681.N	**90% penetrance**
TubGal4	MED10	CG5057	SH01724.N	**wt phenotype**
MTD Gal4	MED11	CG6884	SH01781.N	**50% penetrance**
TubGal4	MED15	CG4184	SH00423.N	**100% undeveloped embryos**
TubGal4	MED16	CG5465	SH01675.N	**10% penetrance**
TubGal4	MED17	CG7957	SH01797.N	**No eggs laid**
TubGal4	MED19	CG5546	SH00843.N	**50% penetrance, many undeveloped embryos**
TubGal4	MED20	CG18780	SH01723.N	**75% penetrance**
TubGal4	MED21	CG17397	SH01783.N	**no eggs laid**
TubGal4	MED22	CG3034	SH01717.N	**99% penetrance**
TubGal4	MED23	CG3695	SH01725.N	**wt phenotype**
TubGal4	MED24	CG7999	SH01798.N	**95% penetrance**
TubGal4	MED25	CG12254	SH00424.N	**<10% penetrance**
TubGal4	MED26	CG1793	TR04124P.1	**wt phenotype**
TubGal4	MED27	CG1245	SH01722.N	**95% undeveloped embryos**
TubGal4	MED28	CG5121	SH00425.N	**100% undeveloped embryos**
TubGal4	MED31	CG1057	SH01720.N	**100% undeveloped embryos, very few eggs laid**
TubGal4	Cdk8	CG10572	SH01410.N2	**100% undeveloped embryos**
TubGal4	CycC	CG7281	SH01782.N	**100% undeveloped embryos**
MTD Gal4	kto	CG8491	SH01796.N	**very few flies hatching, only females**
MTD Gal4	skd	CG9936	SH01787.N	**50% penetrance**

!

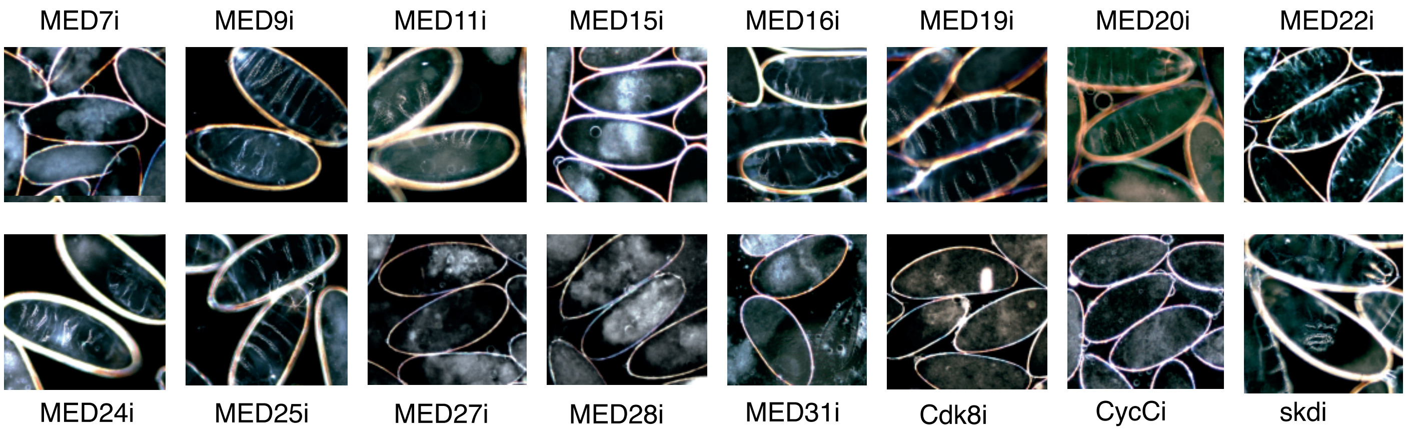

Supplement: S2 Table — The maternal contribution of 26 individual Mediator subunits was knocked-down. Embryos were collected from females containing the maternal α-Tubulin-Gal4-VP16 driver and shmiRNAs targeting Mediator components, and cuticle preparations examined by dark-field microscopy. (PDF) [file pgen.1004971.s007.pdf]
